# Diaqua­bis­{1-[(1*H*-benzimidazol-2-yl)meth­yl]-1*H*-1,2,4-triazole-κ*N* ^4^}bis­(2,4,5-tricarb­oxy­benzoato-κ*O* ^1^)cadmium dihydrate

**DOI:** 10.1107/S1600536812000384

**Published:** 2012-01-11

**Authors:** Lei Zhao, Bingtao Liu, Guanghua Jin, Xiangru Meng

**Affiliations:** a School of Chemical Engineering, Zhengzhou University, Zhengzhou 450001, People’s Republic of China; b School of Environmental and Municipal Engineering, North China Institute of Water Conservancy and Hydroelectric Power, Zhengzhou 450011, People’s Republic of China; c Department of Chemistry, Zhengzhou University, Zhengzhou 450001, People’s Republic of China

## Abstract

In the title complex, [Cd(C_10_H_5_O_8_)_2_(C_10_H_9_N_5_)_2_(H_2_O)_2_]·2H_2_O, the Cd^II^ ion lies on an inversion center and is coordinated by two N atoms from two symmetry-related 1-[(1*H*-benzimidazol-2-yl)meth­yl]-1*H*-1,2,4-triazole ligands and two O atoms from two monodeprotonated 2,4,5-tricarb­oxy­benzoate anions in equatorial positions and by two water O atoms in axial positions, leading to a distorted octa­hedral environment. In the crystal, complex mol­ecules and solvent water mol­ecules are linked through inter­molecular O—H⋯O, O—H⋯N and N—H⋯O hydrogen bonds into a three-dimensional network. Intra­molecular O—H⋯O hydrogen bonds are also present.

## Related literature

For background information on complexes constructed from *N*-heterocyclic ligands and aromatic polycarboxyl­ate anions, see: Braverman *et al.* (2007 ▶); Liu *et al.* (2010 ▶); Prajapati *et al.* (2009 ▶).
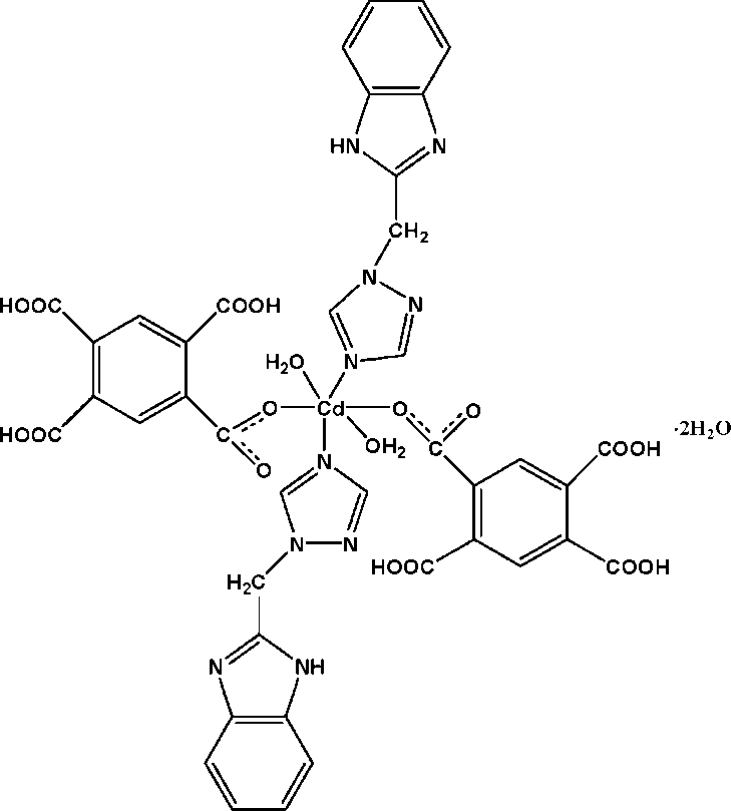


## Experimental

### 

#### Crystal data

[Cd(C_10_H_5_O_8_)_2_(C_10_H_9_N_5_)_2_(H_2_O)_2_]·2H_2_O
*M* *_r_* = 1089.19
Triclinic, 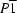

*a* = 7.7005 (15) Å
*b* = 8.6131 (17) Å
*c* = 17.460 (3) Å
α = 75.98 (3)°
β = 82.55 (3)°
γ = 70.60 (3)°
*V* = 1058.2 (3) Å^3^
*Z* = 1
Mo *K*α radiation
μ = 0.62 mm^−1^
*T* = 293 K
0.19 × 0.18 × 0.15 mm

#### Data collection

Rigaku Saturn CCD diffractometer
Absorption correction: multi-scan (*CrystalClear*; Rigaku/MSC, 2004 ▶) *T*_min_ = 0.892, *T*_max_ = 0.913
12522 measured reflections
4987 independent reflections
4758 reflections with *I* > 2σ(*I*)
*R*_int_ = 0.023

#### Refinement

*R*[*F* ^2^ > 2σ(*F* ^2^)] = 0.033
*wR*(*F* ^2^) = 0.084
*S* = 1.05
4987 reflections
322 parameters
H-atom parameters constrained
Δρ_max_ = 0.66 e Å^−3^
Δρ_min_ = −0.65 e Å^−3^

### 

Data collection: *CrystalClear* (Rigaku/MSC, 2004 ▶); cell refinement: *CrystalClear*; data reduction: *CrystalClear*; program(s) used to solve structure: *SHELXTL* (Sheldrick, 2008 ▶); program(s) used to refine structure: *SHELXTL*; molecular graphics: *SHELXTL*; software used to prepare material for publication: *publCIF* (Westrip, 2010 ▶).

## Supplementary Material

Crystal structure: contains datablock(s) global, I. DOI: 10.1107/S1600536812000384/wm2579sup1.cif

Structure factors: contains datablock(s) I. DOI: 10.1107/S1600536812000384/wm2579Isup2.hkl

Additional supplementary materials:  crystallographic information; 3D view; checkCIF report

## Figures and Tables

**Table 1:** Selected bond lengths (Å)

Cd1—O1	2.2933 (15)
Cd1—O9	2.3155 (17)
Cd1—N1	2.358 (2)

**Table 2:** Hydrogen-bond geometry (Å, °)

*D*—H⋯*A*	*D*—H	H⋯*A*	*D*⋯*A*	*D*—H⋯*A*
O3—H3⋯O1	0.85	2.19	3.001 (3)	160
O5—H5⋯O6^i^	0.85	1.77	2.611 (3)	168
O7—H7⋯O4^ii^	0.85	1.66	2.465 (2)	156
O9—H9*A*⋯O8^iii^	0.85	2.01	2.844 (2)	167
O9—H9*B*⋯O2	0.85	1.94	2.688 (2)	147
O10—H10*A*⋯O8^iv^	0.85	2.05	2.887 (3)	169
O10—H10*B*⋯N4	0.85	2.21	2.704 (3)	117
N5—H5*A*⋯O8^v^	0.86	2.13	2.922 (3)	153

Symmetry codes: (i) 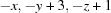; (ii) 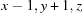; (iii) 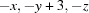; (iv) 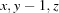; (v) 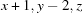.

**Fig. 1.**
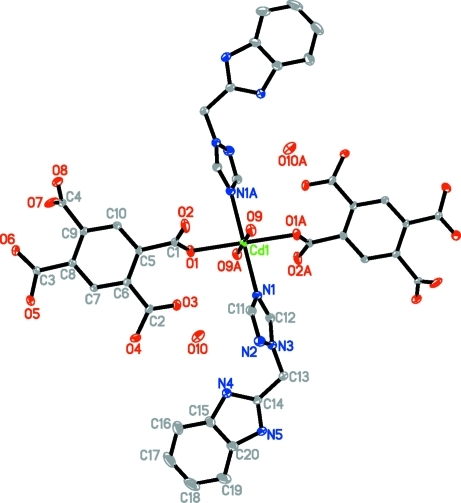
View of the molecular struicture of the title complex showing the atom labelling with displacement ellipsoids drawn at the 30% probability level. [Symmetry code A) -x, -y + 2, -z.]

**Fig. 2.**
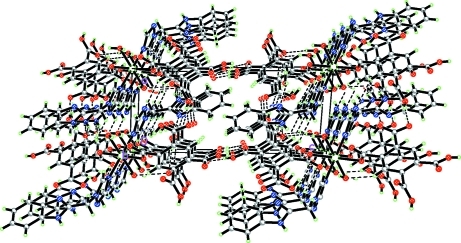
View of the crystal packing of the title complex, showing the three-dimensional structure stabilized by numerous hydrogen bonds.

## supplementary crystallographic information

### Comment

A large number of Cd^II^ complexes constructed from N-heterocyclic and aromatic polycarboxylate ligands have been synthesized since Cd^II^ is able to coordinate simultaneously to both oxygen-containing and nitrogen-containing ligands. Some of the final products exhibit useful functional properties (Braverman *et al.*, 2007; Liu *et al.*, 2010; Prajapati *et al.*, 2009). In order to further explore such compounds with new structures, we selected 1-((1*H*-benzimidazol-1-yl)methyl)-1*H*-1,2,4-triazole and 1,2,4,5-benzenetetracarboxylic acid as educts to self-assemble with Cd(NO_3_)_2_ and obtained the title complex, {[Cd(C_10_H_5_O_8_)_2_(C_10_H_9_N_5_)_2_(H_2_O)_2_] (H_2_O)_2_}, the crystal structure of which is reported herein.

The Cd^II^ ion lies on an inversion center and displays a slightly distorted octahedral geometry defined by atoms O1, O1A, N1, N1A from two 1-((1*H*-benzimidazol-1-yl)methyl)-1*H*-1,2,4-triazole ligands and two monodeprotonated 1,2,4,5-benzenetetracarboxylic acid anions in equatorial positions, and by atoms O9, O9A from water molecules in axial positions (Fig. 1). Intramolecular O—H···O hydrogen bonds between the carboxyl/carboxylate groups and between coordinating water molecules and carboxylate O atoms stabilize the molecular configuration, whereas O—H···O, O—H···N and N—H···O hydrogen bonds between carboxyl/carboxylate groups, between coordinating water molecules and carboxylate O atoms, between solvent water molecules and carboxylate O atoms, between imidazole groups and carboxylate O atoms and between solvent water molecules and imidazole N atoms of adjacent molecules consolidate the crystal packing (Fig. 2).

### Experimental

A mixture of Cd(NO_3_)_2_ (0.05 mmol), 1-((1*H*-benzimidazol-1-yl)methyl)-1*H*-1,2,4-triazole (0.05 mmol) 1,2,4,5-benzenetetracarboxylic acid (0.05 mmol), methanol (2 ml) and water (8 ml) was placed in a 25 ml Teflon-lined stainless steel vessel and heated at 393 K for 72 h, then cooled to room temperature. Colourless crystals were obtained from the evaporated filtrate and dried in air.

### Refinement

H atoms bound to C atoms were positioned geometrically and refined as riding atoms, with C—H = 0.93 (aromatic) Å and 0.97 (CH_2_) Å. H atoms bound to N and O atoms were found from difference maps and refined with ditance restraints of N—H = 0.86 Å and O—H = 0.85 Å. All H atoms were refined with *U*_iso_(H) = 1.2 *U*_eq_(C,*N*,*O*).

### Crystal data

**Table:**

[Cd(C_10_H_5_O_8_)_2_(C_10_H_9_N_5_)_2_(H_2_O)_2_]·2H_2_O	*Z* = 1
*M**_r_* = 1089.19	*F*(000) = 554
Triclinic, *P*1	*D*_x_ = 1.709 Mg m^−^^3^
Hall symbol: -P 1	Mo *K*α radiation, λ = 0.71073 Å
*a* = 7.7005 (15) Å	Cell parameters from 3785 reflections
*b* = 8.6131 (17) Å	θ = 2.4–27.9°
*c* = 17.460 (3) Å	µ = 0.62 mm^−^^1^
α = 75.98 (3)°	*T* = 293 K
β = 82.55 (3)°	Prism, colourless
γ = 70.60 (3)°	0.19 × 0.18 × 0.15 mm
*V* = 1058.2 (3) Å^3^	

### Data collection

**Table:**

Rigaku Saturn CCD diffractometer	4987 independent reflections
Radiation source: fine-focus sealed tube	4758 reflections with *I* > 2σ(*I*)
graphite	*R*_int_ = 0.023
Detector resolution: 28.5714 pixels mm^-1^	θ_max_ = 27.9°, θ_min_ = 2.4°
ω scans	*h* = −10→10
Absorption correction: multi-scan (*CrystalClear*; Rigaku/MSC, 2004)	*k* = −11→11
*T*_min_ = 0.892, *T*_max_ = 0.913	*l* = −22→21
12522 measured reflections	

### Refinement

**Table:**

Refinement on *F*^2^	Primary atom site location: structure-invariant direct methods
Least-squares matrix: full	Secondary atom site location: difference Fourier map
*R*[*F*^2^ > 2σ(*F*^2^)] = 0.033	Hydrogen site location: inferred from neighbouring sites
*wR*(*F*^2^) = 0.084	H-atom parameters constrained
*S* = 1.05	*w* = 1/[σ^2^(*F*_o_^2^) + (0.0457*P*)^2^ + 0.6556*P*] where *P* = (*F*_o_^2^ + 2*F*_c_^2^)/3
4987 reflections	(Δ/σ)_max_ < 0.001
322 parameters	Δρ_max_ = 0.66 e Å^−^^3^
0 restraints	Δρ_min_ = −0.65 e Å^−^^3^

### Special details

**Table:**

Geometry. All e.s.d.'s (except the e.s.d. in the dihedral angle between two l.s. planes) are estimated using the full covariance matrix. The cell e.s.d.'s are taken into account individually in the estimation of e.s.d.'s in distances, angles and torsion angles; correlations between e.s.d.'s in cell parameters are only used when they are defined by crystal symmetry. An approximate (isotropic) treatment of cell e.s.d.'s is used for estimating e.s.d.'s involving l.s. planes.
Refinement. Refinement of *F*^2^ against ALL reflections. The weighted *R*-factor *wR* and goodness of fit *S* are based on *F*^2^, conventional *R*-factors *R* are based on *F*, with *F* set to zero for negative *F*^2^. The threshold expression of *F*^2^ > σ(*F*^2^) is used only for calculating *R*-factors(gt) *etc*. and is not relevant to the choice of reflections for refinement. *R*-factors based on *F*^2^ are statistically about twice as large as those based on *F*, and *R*- factors based on ALL data will be even larger.

### Fractional atomic coordinates and isotropic or equivalent isotropic displacement parameters (Å^2^)

**Table:**

	*x*	*y*	*z*	*U*_iso_*/*U*_eq_	
Cd1	0.0000	1.0000	0.0000	0.01995 (7)	
N1	0.2410 (2)	0.7537 (2)	0.04738 (11)	0.0250 (4)	
N2	0.5217 (3)	0.6030 (2)	0.09222 (12)	0.0295 (4)	
N3	0.4177 (2)	0.5005 (2)	0.09340 (11)	0.0238 (4)	
N4	0.4526 (3)	0.3824 (2)	0.25900 (11)	0.0269 (4)	
N5	0.6329 (3)	0.1335 (2)	0.24746 (12)	0.0321 (4)	
H5A	0.6930	0.0485	0.2268	0.038*	
O1	−0.0272 (2)	1.1045 (2)	0.11147 (9)	0.0285 (3)	
O2	0.1184 (3)	1.2920 (2)	0.05454 (10)	0.0385 (4)	
O3	0.3379 (3)	0.9741 (2)	0.18617 (10)	0.0397 (4)	
H3	0.2496	0.9980	0.1563	0.048*	
O4	0.3395 (3)	0.9197 (2)	0.31666 (10)	0.0389 (4)	
O5	0.0260 (4)	1.3218 (2)	0.46604 (11)	0.0573 (6)	
H5	0.0324	1.3638	0.5046	0.069*	
O6	−0.0760 (3)	1.5906 (2)	0.40745 (11)	0.0558 (6)	
O7	−0.3986 (2)	1.6650 (2)	0.30619 (11)	0.0410 (4)	
H7	−0.4707	1.7657	0.3002	0.049*	
O8	−0.2047 (2)	1.78648 (19)	0.22713 (10)	0.0308 (3)	
O9	0.2285 (2)	1.1153 (2)	−0.06019 (9)	0.0309 (4)	
H9A	0.2325	1.1543	−0.1098	0.037*	
H9B	0.2115	1.1975	−0.0383	0.037*	
O10	0.1906 (3)	0.6873 (2)	0.23452 (12)	0.0431 (4)	
H10A	0.0737	0.7278	0.2353	0.052*	
H10B	0.1948	0.5903	0.2295	0.052*	
C1	0.0434 (3)	1.2165 (3)	0.11195 (12)	0.0216 (4)	
C2	0.2772 (3)	1.0108 (3)	0.24903 (13)	0.0231 (4)	
C3	−0.0278 (3)	1.4403 (3)	0.40573 (13)	0.0293 (5)	
C4	−0.2513 (3)	1.6628 (3)	0.26677 (12)	0.0233 (4)	
C5	0.0265 (3)	1.2716 (2)	0.18958 (11)	0.0189 (4)	
C6	0.1271 (3)	1.1709 (2)	0.25503 (12)	0.0200 (4)	
C7	0.0996 (3)	1.2271 (3)	0.32503 (12)	0.0230 (4)	
H7A	0.1636	1.1578	0.3688	0.028*	
C8	−0.0216 (3)	1.3844 (3)	0.33102 (12)	0.0219 (4)	
C9	−0.1177 (3)	1.4889 (2)	0.26518 (12)	0.0201 (4)	
C10	−0.0942 (3)	1.4299 (3)	0.19571 (12)	0.0205 (4)	
H10	−0.1609	1.4980	0.1524	0.025*	
C11	0.4094 (3)	0.7527 (3)	0.06434 (13)	0.0274 (4)	
H11	0.4418	0.8504	0.0567	0.033*	
C12	0.2522 (3)	0.5920 (3)	0.06641 (13)	0.0248 (4)	
H12	0.1590	0.5491	0.0616	0.030*	
C13	0.4912 (3)	0.3198 (3)	0.12359 (13)	0.0265 (4)	
H13A	0.6060	0.2753	0.0944	0.032*	
H13B	0.4048	0.2659	0.1153	0.032*	
C14	0.5248 (3)	0.2790 (3)	0.20956 (13)	0.0243 (4)	
C15	0.5184 (4)	0.2989 (3)	0.33352 (14)	0.0320 (5)	
C16	0.4865 (5)	0.3505 (4)	0.40519 (16)	0.0488 (7)	
H16	0.4082	0.4564	0.4104	0.059*	
C17	0.5784 (7)	0.2342 (5)	0.46774 (18)	0.0706 (11)	
H17	0.5617	0.2624	0.5169	0.085*	
C18	0.6953 (7)	0.0761 (5)	0.4597 (2)	0.0862 (15)	
H18	0.7548	0.0026	0.5038	0.103*	
C19	0.7268 (6)	0.0237 (4)	0.3894 (2)	0.0657 (10)	
H19	0.8050	−0.0824	0.3845	0.079*	
C20	0.6336 (4)	0.1404 (3)	0.32615 (15)	0.0368 (6)	

### Atomic displacement parameters (Å^2^)

**Table:**

	*U*^11^	*U*^22^	*U*^33^	*U*^12^	*U*^13^	*U*^23^
Cd1	0.02136 (11)	0.02025 (12)	0.01892 (11)	−0.00516 (8)	−0.00346 (7)	−0.00608 (8)
N1	0.0255 (9)	0.0240 (9)	0.0242 (9)	−0.0046 (7)	−0.0072 (7)	−0.0039 (7)
N2	0.0256 (9)	0.0314 (10)	0.0325 (10)	−0.0098 (8)	−0.0068 (8)	−0.0045 (8)
N3	0.0241 (9)	0.0229 (9)	0.0233 (9)	−0.0048 (7)	−0.0042 (7)	−0.0049 (7)
N4	0.0312 (10)	0.0244 (9)	0.0232 (9)	−0.0056 (8)	−0.0024 (7)	−0.0054 (7)
N5	0.0381 (11)	0.0205 (9)	0.0326 (10)	0.0016 (8)	−0.0093 (9)	−0.0078 (8)
O1	0.0361 (8)	0.0321 (8)	0.0249 (8)	−0.0147 (7)	−0.0028 (6)	−0.0138 (7)
O2	0.0639 (12)	0.0396 (10)	0.0224 (8)	−0.0281 (9)	0.0055 (8)	−0.0134 (7)
O3	0.0416 (10)	0.0385 (10)	0.0295 (9)	0.0115 (8)	−0.0087 (7)	−0.0199 (8)
O4	0.0429 (10)	0.0274 (9)	0.0284 (9)	0.0143 (7)	−0.0057 (7)	−0.0062 (7)
O5	0.1076 (18)	0.0305 (10)	0.0227 (9)	0.0044 (11)	−0.0244 (10)	−0.0108 (8)
O6	0.1015 (18)	0.0260 (9)	0.0304 (9)	0.0067 (10)	−0.0248 (11)	−0.0153 (8)
O7	0.0338 (9)	0.0192 (8)	0.0515 (11)	0.0072 (7)	0.0105 (8)	−0.0020 (8)
O8	0.0371 (9)	0.0181 (7)	0.0327 (8)	−0.0030 (7)	−0.0017 (7)	−0.0049 (6)
O9	0.0377 (9)	0.0342 (9)	0.0268 (8)	−0.0191 (7)	0.0033 (7)	−0.0087 (7)
O10	0.0361 (10)	0.0288 (9)	0.0634 (13)	−0.0049 (8)	−0.0022 (9)	−0.0154 (9)
C1	0.0242 (10)	0.0206 (10)	0.0198 (9)	−0.0014 (8)	−0.0067 (8)	−0.0086 (8)
C2	0.0209 (9)	0.0193 (10)	0.0287 (11)	0.0001 (8)	−0.0045 (8)	−0.0116 (8)
C3	0.0406 (13)	0.0221 (10)	0.0194 (10)	0.0029 (9)	−0.0063 (9)	−0.0089 (8)
C4	0.0281 (10)	0.0173 (10)	0.0191 (9)	0.0024 (8)	−0.0060 (8)	−0.0051 (8)
C5	0.0202 (9)	0.0196 (9)	0.0186 (9)	−0.0050 (8)	−0.0025 (7)	−0.0081 (8)
C6	0.0209 (9)	0.0171 (9)	0.0214 (9)	−0.0013 (8)	−0.0038 (7)	−0.0078 (8)
C7	0.0269 (10)	0.0188 (10)	0.0190 (9)	0.0018 (8)	−0.0075 (8)	−0.0056 (8)
C8	0.0271 (10)	0.0173 (9)	0.0181 (9)	0.0006 (8)	−0.0046 (8)	−0.0067 (8)
C9	0.0205 (9)	0.0166 (9)	0.0209 (9)	−0.0003 (7)	−0.0026 (7)	−0.0065 (8)
C10	0.0217 (9)	0.0186 (9)	0.0190 (9)	−0.0009 (8)	−0.0075 (7)	−0.0040 (8)
C11	0.0275 (11)	0.0282 (11)	0.0282 (11)	−0.0101 (9)	−0.0066 (9)	−0.0045 (9)
C12	0.0246 (10)	0.0254 (10)	0.0246 (10)	−0.0064 (8)	−0.0057 (8)	−0.0055 (8)
C13	0.0280 (11)	0.0212 (10)	0.0263 (11)	−0.0009 (8)	−0.0046 (8)	−0.0060 (8)
C14	0.0248 (10)	0.0207 (10)	0.0258 (10)	−0.0041 (8)	−0.0039 (8)	−0.0050 (8)
C15	0.0439 (14)	0.0280 (12)	0.0247 (11)	−0.0144 (10)	−0.0037 (10)	−0.0015 (9)
C16	0.080 (2)	0.0409 (15)	0.0287 (13)	−0.0232 (15)	0.0006 (13)	−0.0085 (12)
C17	0.132 (4)	0.061 (2)	0.0241 (14)	−0.038 (2)	−0.0179 (18)	−0.0009 (14)
C18	0.154 (4)	0.055 (2)	0.0416 (19)	−0.024 (3)	−0.048 (2)	0.0148 (16)
C19	0.102 (3)	0.0344 (16)	0.0494 (18)	−0.0056 (17)	−0.0356 (19)	0.0053 (14)
C20	0.0507 (15)	0.0271 (12)	0.0311 (12)	−0.0107 (11)	−0.0137 (11)	0.0008 (10)

### Geometric parameters (Å, °)

**Table:**

Cd1—O1^i^	2.2933 (15)	O10—H10A	0.8500
Cd1—O1	2.2933 (15)	O10—H10B	0.8500
Cd1—O9^i^	2.3154 (17)	C1—C5	1.518 (3)
Cd1—O9	2.3155 (17)	C2—C6	1.493 (3)
Cd1—N1^i^	2.358 (2)	C3—C8	1.486 (3)
Cd1—N1	2.358 (2)	C4—C9	1.514 (3)
N1—C12	1.327 (3)	C5—C10	1.392 (3)
N1—C11	1.364 (3)	C5—C6	1.400 (3)
N2—C11	1.308 (3)	C6—C7	1.387 (3)
N2—N3	1.371 (3)	C7—C8	1.386 (3)
N3—C12	1.332 (3)	C7—H7A	0.9300
N3—C13	1.454 (3)	C8—C9	1.399 (3)
N4—C14	1.329 (3)	C9—C10	1.394 (3)
N4—C15	1.394 (3)	C10—H10	0.9300
N5—C14	1.323 (3)	C11—H11	0.9300
N5—C20	1.390 (3)	C12—H12	0.9300
N5—H5A	0.8600	C13—C14	1.493 (3)
O1—C1	1.257 (3)	C13—H13A	0.9700
O2—C1	1.243 (3)	C13—H13B	0.9700
O3—C2	1.208 (3)	C15—C20	1.385 (4)
O3—H3	0.8501	C15—C16	1.393 (4)
O4—C2	1.299 (3)	C16—C17	1.377 (4)
O5—C3	1.278 (3)	C16—H16	0.9300
O5—H5	0.8504	C17—C18	1.389 (6)
O6—C3	1.229 (3)	C17—H17	0.9300
O7—C4	1.245 (3)	C18—C19	1.374 (5)
O7—H7	0.8500	C18—H18	0.9300
O8—C4	1.255 (3)	C19—C20	1.390 (4)
O9—H9A	0.8501	C19—H19	0.9300
O9—H9B	0.8499		
			
O1^i^—Cd1—O1	180.0	C7—C6—C5	119.85 (18)
O1^i^—Cd1—O9^i^	93.80 (6)	C7—C6—C2	118.63 (18)
O1—Cd1—O9^i^	86.20 (6)	C5—C6—C2	121.27 (18)
O1^i^—Cd1—O9	86.20 (6)	C8—C7—C6	121.30 (19)
O1—Cd1—O9	93.80 (6)	C8—C7—H7A	119.3
O9^i^—Cd1—O9	180.000 (1)	C6—C7—H7A	119.4
O1^i^—Cd1—N1^i^	93.81 (7)	C7—C8—C9	119.42 (18)
O1—Cd1—N1^i^	86.19 (7)	C7—C8—C3	117.30 (18)
O9^i^—Cd1—N1^i^	86.47 (7)	C9—C8—C3	122.97 (18)
O9—Cd1—N1^i^	93.53 (7)	C10—C9—C8	119.14 (18)
O1^i^—Cd1—N1	86.19 (7)	C10—C9—C4	118.22 (18)
O1—Cd1—N1	93.81 (7)	C8—C9—C4	122.62 (18)
O9^i^—Cd1—N1	93.53 (7)	C5—C10—C9	121.59 (18)
O9—Cd1—N1	86.47 (7)	C5—C10—H10	119.2
N1^i^—Cd1—N1	180.00 (9)	C9—C10—H10	119.2
C12—N1—C11	103.37 (18)	N2—C11—N1	114.5 (2)
C12—N1—Cd1	132.41 (15)	N2—C11—H11	122.7
C11—N1—Cd1	124.19 (15)	N1—C11—H11	122.7
C11—N2—N3	102.43 (17)	N1—C12—N3	109.49 (19)
C12—N3—N2	110.17 (18)	N1—C12—H12	125.3
C12—N3—C13	129.49 (19)	N3—C12—H12	125.3
N2—N3—C13	120.31 (17)	N3—C13—C14	111.69 (18)
C14—N4—C15	108.15 (19)	N3—C13—H13A	109.3
C14—N5—C20	108.8 (2)	C14—C13—H13A	109.3
C14—N5—H5A	125.6	N3—C13—H13B	109.3
C20—N5—H5A	125.6	C14—C13—H13B	109.3
C1—O1—Cd1	120.44 (14)	H13A—C13—H13B	107.9
C2—O3—H3	109.5	N5—C14—N4	110.1 (2)
C3—O5—H5	109.4	N5—C14—C13	124.4 (2)
C4—O7—H7	109.5	N4—C14—C13	125.46 (19)
Cd1—O9—H9A	120.9	C20—C15—C16	122.0 (2)
Cd1—O9—H9B	103.5	C20—C15—N4	106.7 (2)
H9A—O9—H9B	106.5	C16—C15—N4	131.3 (2)
H10A—O10—H10B	95.5	C17—C16—C15	115.6 (3)
O2—C1—O1	126.63 (19)	C17—C16—H16	122.2
O2—C1—C5	116.63 (18)	C15—C16—H16	122.2
O1—C1—C5	116.65 (18)	C16—C17—C18	122.1 (3)
O3—C2—O4	123.89 (19)	C16—C17—H17	118.9
O3—C2—C6	122.1 (2)	C18—C17—H17	118.9
O4—C2—C6	113.95 (18)	C19—C18—C17	122.8 (3)
O6—C3—O5	123.4 (2)	C19—C18—H18	118.6
O6—C3—C8	121.3 (2)	C17—C18—H18	118.6
O5—C3—C8	115.20 (19)	C18—C19—C20	115.4 (3)
O7—C4—O8	127.6 (2)	C18—C19—H19	122.3
O7—C4—C9	115.04 (19)	C20—C19—H19	122.3
O8—C4—C9	117.30 (19)	C15—C20—N5	106.2 (2)
C10—C5—C6	118.63 (18)	C15—C20—C19	122.2 (3)
C10—C5—C1	118.31 (17)	N5—C20—C19	131.6 (3)
C6—C5—C1	123.07 (17)		
			
O1^i^—Cd1—N1—C12	−65.3 (2)	C7—C8—C9—C4	−179.5 (2)
O1—Cd1—N1—C12	114.7 (2)	C3—C8—C9—C4	−6.1 (3)
O9^i^—Cd1—N1—C12	28.3 (2)	O7—C4—C9—C10	107.0 (2)
O9—Cd1—N1—C12	−151.7 (2)	O8—C4—C9—C10	−71.0 (3)
N1^i^—Cd1—N1—C12	147 (100)	O7—C4—C9—C8	−71.2 (3)
O1^i^—Cd1—N1—C11	117.24 (18)	O8—C4—C9—C8	110.8 (2)
O1—Cd1—N1—C11	−62.76 (18)	C6—C5—C10—C9	−0.5 (3)
O9^i^—Cd1—N1—C11	−149.18 (17)	C1—C5—C10—C9	179.71 (19)
O9—Cd1—N1—C11	30.82 (17)	C8—C9—C10—C5	−1.9 (3)
N1^i^—Cd1—N1—C11	−30 (100)	C4—C9—C10—C5	179.80 (19)
C11—N2—N3—C12	0.3 (2)	N3—N2—C11—N1	−0.1 (3)
C11—N2—N3—C13	−177.99 (19)	C12—N1—C11—N2	−0.1 (3)
O1^i^—Cd1—O1—C1	44 (100)	Cd1—N1—C11—N2	178.02 (15)
O9^i^—Cd1—O1—C1	−166.06 (16)	C11—N1—C12—N3	0.2 (2)
O9—Cd1—O1—C1	13.94 (16)	Cd1—N1—C12—N3	−177.62 (14)
N1^i^—Cd1—O1—C1	−79.36 (16)	N2—N3—C12—N1	−0.3 (3)
N1—Cd1—O1—C1	100.64 (16)	C13—N3—C12—N1	177.7 (2)
Cd1—O1—C1—O2	6.3 (3)	C12—N3—C13—C14	−113.5 (2)
Cd1—O1—C1—C5	−177.03 (12)	N2—N3—C13—C14	64.4 (3)
O2—C1—C5—C10	69.1 (3)	C20—N5—C14—N4	−0.3 (3)
O1—C1—C5—C10	−107.8 (2)	C20—N5—C14—C13	179.3 (2)
O2—C1—C5—C6	−110.6 (2)	C15—N4—C14—N5	0.2 (3)
O1—C1—C5—C6	72.4 (3)	C15—N4—C14—C13	−179.4 (2)
C10—C5—C6—C7	2.6 (3)	N3—C13—C14—N5	−161.6 (2)
C1—C5—C6—C7	−177.66 (19)	N3—C13—C14—N4	18.0 (3)
C10—C5—C6—C2	−171.46 (18)	C14—N4—C15—C20	0.0 (3)
C1—C5—C6—C2	8.3 (3)	C14—N4—C15—C16	179.6 (3)
O3—C2—C6—C7	−162.3 (2)	C20—C15—C16—C17	0.6 (4)
O4—C2—C6—C7	15.3 (3)	N4—C15—C16—C17	−178.9 (3)
O3—C2—C6—C5	11.8 (3)	C15—C16—C17—C18	0.1 (6)
O4—C2—C6—C5	−170.64 (19)	C16—C17—C18—C19	−0.5 (7)
C5—C6—C7—C8	−2.3 (3)	C17—C18—C19—C20	0.2 (7)
C2—C6—C7—C8	171.9 (2)	C16—C15—C20—N5	−179.8 (2)
C6—C7—C8—C9	−0.2 (3)	N4—C15—C20—N5	−0.2 (3)
C6—C7—C8—C3	−174.0 (2)	C16—C15—C20—C19	−1.0 (5)
O6—C3—C8—C7	154.1 (3)	N4—C15—C20—C19	178.6 (3)
O5—C3—C8—C7	−23.7 (3)	C14—N5—C20—C15	0.3 (3)
O6—C3—C8—C9	−19.5 (4)	C14—N5—C20—C19	−178.4 (3)
O5—C3—C8—C9	162.7 (2)	C18—C19—C20—C15	0.5 (5)
C7—C8—C9—C10	2.2 (3)	C18—C19—C20—N5	179.1 (4)
C3—C8—C9—C10	175.7 (2)		

Symmetry codes: (i) −*x*, −*y*+2, −*z*.

### Hydrogen-bond geometry (Å, °)

**Table:**

*D*—H···*A*	*D*—H	H···*A*	*D*···*A*	*D*—H···*A*
O3—H3···O1	0.85	2.19	3.001 (3)	160
O5—H5···O6^ii^	0.85	1.77	2.611 (3)	168
O7—H7···O4^iii^	0.85	1.66	2.465 (2)	156
O9—H9A···O8^iv^	0.85	2.01	2.844 (2)	167
O9—H9B···O2	0.85	1.94	2.688 (2)	147
O10—H10A···O8^v^	0.85	2.05	2.887 (3)	169
O10—H10B···N4	0.85	2.21	2.704 (3)	117
N5—H5A···O8^vi^	0.86	2.13	2.922 (3)	153

Symmetry codes: (ii) −*x*, −*y*+3, −*z*+1; (iii) *x*−1, *y*+1, *z*; (iv) −*x*, −*y*+3, −*z*; (v) *x*, *y*−1, *z*; (vi) *x*+1, *y*−2, *z*.
